# Strategies for caffeine administration in preterm infants: route, dosing, monitoring, and adjunctive interventions - a systematic review

**DOI:** 10.1186/s13052-026-02344-0

**Published:** 2026-09-21

**Authors:** Alaa A. M. Osman, Chiara Russo, Maria Rojas-Reyes, Franciszek Borys, Michelle Fiander, Roger Soll, Matteo Bruschettini, Greta Sibrecht

**Affiliations:** 1 Department of Clinical Pharmacy and Pharmacy Practice, Faculty of Pharmacy, University of Gezira, Wad Madani, Sudan; 2 Department of Neuroscience, Rehabilitation, Ophthalmology, Genetics and Maternal and Child Sciences, University of Genova, Genova, Italy; 3 Institut de Recerca Sant Pau (IR SANT PAU), Barcelona, Spain; 4 II Department of Neonatology, Poznan University of Medical Sciences, Poznan, Poland; 5 Cochrane Neonatal Group, Burlington, VT USA; 6 Cochrane Sweden, Department of Research, Development, Education and Innovation, Lund University, Skåne University Hospital, Lund, Sweden

**Keywords:** Caffeine, Preterm infants, Apnea of prematurity, Dosing strategies, Surfactant, Systematic review, Oral administration, Dosing frequency, Dose modification

## Abstract

**Introduction:**

Caffeine is widely used in preterm infants, yet the optimal administration strategy remains unclear. This systematic review aims to evaluate different caffeine administration approaches, including route, frequency, dose modifications, and co-administration with surfactant in preterm infants.

**Methods:**

We searched three databases and two trial registries up to October 2024. Data collection and analysis followed Cochrane methodology; certainty of evidence was assessed using GRADE; and reporting followed PRISMA.

**Results:**

Three RCTs were identified, two provided data for analyses: one study (38 infants) on route of administration (intravenous versus oral caffeine) and one study (40 infants) on frequency of administration (once versus twice daily). The evidence is very uncertain about the effects of intravenous versus oral administration on: mortality (RR 0.14, 95% CI 0.01–2.59); mechanical ventilation duration (MD 1.00, 95% CI -8.05 to 10.05); hospital stay (MD 8.00, 95%CI -5.35-21.35); severe intraventricular hemorrhage (RR 1.00, 95%CI 0.29–3.43); and chronic lung disease (RR 5.00, 95%CI 0.26–97.70). The evidence is very uncertain about the effects of twice-daily versus once-daily caffeine on: mortality (RR 1.00, 95%CI 0.29–3.45); ventilation duration (MD 0.81, 95%CI -7.56-9.18); and hospital stay (MD -2.70, 95%CI -8.12-2.72). We found no studies on caffeine dose modifications and co-administration with surfactant. Two trials on adjusting caffeine dose and route of administration are ongoing.

**Conclusion:**

Although caffeine clearly benefits preterm infants, evidence guiding optimal administration strategies, such as route, frequency, and dose modifications, is limited. High-quality studies are urgently needed to inform clinical practice and guidelines.

**Supplementary Information:**

The online version contains supplementary material available at https://doi.org/10.1186/s13052-026-02344-0.

## Introduction

Preterm infants have a higher risk of mortality, which increases as gestational age (GA) decreases [1]. Apnea of prematurity (AOP) is a common complication caused by an immature central nervous system, leading to reduced ventilatory drive [2]. Recurrent hypoxic episodes may increase the need of extubation failure and prolong the need for respiratory support [3]. In extremely preterm infants, prolonged apnea episodes elevate the risk of severe bronchopulmonary dysplasia (BPD) [4]. Additionally, oxidative stress from AOP increases the likelihood of neurodevelopmental impairment [5].

Among methylxanthines, caffeine is the safest and most effective for facilitating extubation in mechanically ventilated infants and treating or preventing AOP [6]. It stimulates breathing, improves diaphragmatic function by blocking adenosine A1 and A2a receptors in the brainstem [6]and also has antioxidative [7] and diuretic effects [8].

Caffeine has a favorable pharmacokinetic profile in preterm infants. It has high bioavailability, allowing for both oral and intravenous administration, a long half-life (over 100 h) supporting once-daily dosing, and a wide therapeutic index, permitting high doses without significant harm or the need for routine serum monitoring [9, 10].

Caffeine citrate is commonly administered at a loading dose of 20 mg/kg (10 mg/kg caffeine base) followed by a maintenance dose of 5–10 mg/kg/day (2.5–5 mg/kg caffeine base) [11]. This regimen maintains plasma levels in the therapeutic range (8–20 mg/L) to effectively reduce apnea [12, 13]. Preterm infants tolerate higher doses well-even at plasma levels ≥ 70 mg/L [14], which allows dose adjustments in those with poor clinical response.

Systematic reviews [11, 14] suggest that higher doses may provide greater benefits than standard doses. Caffeine clearance increases with postnatal age, from 1 mL/min/kg on the first day to 12 mL/min/kg at 45 days [10], thus reducing its half-life over time, and potentially affecting treatment response. Therefore, adjusting doses as the infant matures or splitting the total daily dose may be beneficial.

Optimizing caffeine administration could improve outcomes, reduce long-term complications, and ease the healthcare burden associated with prematurity. However, despite its routine use in neonatal care, standardized protocols for optimal caffeine administration are lacking.

## Objectives

To evaluate the benefits and harms of different strategies for caffeine administration in preterm infants, based on route (intravenous versus oral); frequency (twice versus once-daily) of administration; dose modification (depending on either poor clinical response, postnatal age or caffeine blood levels); or co-administration of caffeine and surfactant. These research and clinical questions have been identified following a priority exercise to develop national guidelines on the use of caffeine in preterm infants

## Methods

The review was conducted following Cochrane methodology, per the Cochrane Handbook for Systematic Reviews of Interventions [15]. Risk of bias in included trials was assessed using the Cochrane Risk of Bias tool, version 2.0 (RoB 2.0) Excel tool [16]; and certainty of evidence was assessed using the GRADE (Grading of Recommendations, Assessment, Development, and Evaluation) approach [17]. The review was reported following the Preferred Reporting Items for Systematic Reviews and Meta-Analyses (PRISMA) *guidelines* [18]. The protocol was registered in the International Prospective Register of Systematic Reviews (PROSPERO; CRD42024608091). A post-hoc decision was made to revise the comparison regarding adjunct therapy to caffeine - surfactant, as it was found to be misleading in the initial version.

### Criteria for considering studies for this review

#### Types of studies

We included parallel groups randomized controlled trials (RCTs), quasi-RCTs (those where allocation is decided by an approximation of randomization, e.g., by patient ID number) and cluster-RCTs.

#### Participants

We included preterm infants born at < 37 weeks’ gestation, at a postmenstrual age (PMA) up to 44 weeks and 0 days, who received caffeine for any indication.

#### Interventions and comparators

Caffeine dosing:

- Route of administration: intravenous versus oral administration of the initial caffeine dose
- Frequency of administration: twice-daily (split dose) caffeine administration versus once-daily caffeine administration
- Dose modification:
  - Higher versus standard dose caffeine in preterm infants with poor clinical response
  - Adjusting caffeine dose as the infant grows versus maintaining the same dose over time
  - Adjusting caffeine dose based on caffeine blood levels

Adjunct therapy to caffeine—surfactant:

- Delivery room administration of caffeine and surfactant versus surfactant alone
- Early combined administration of caffeine and surfactant versus surfactant alone

#### Outcome measures

Outcomes are detailed below. To forestall reporting bias, studies which planned to collect outcome data of interest in this review were included even if they had no data on the outcome.

#### Critical outcomes

1. All-cause mortality until hospital discharge.
2. Chronic lung disease (CLD) assessed at 36 weeks’ PMA, defined as need for oxygen or respiratory support.
3. Any adverse event leading to caffeine cessation until hospital discharge.

#### Important outcomes

1. Duration of mechanical ventilation (MV) until hospital discharge (days).
2. Duration of hospital stay until hospital discharge (days).
3. Apnea: number of infants with at least one episode (defined as interruption of breathing for > 20 s) until hospital discharge.
4. Intermittent hypoxemia number of infants with at least one episode until hospital discharge.
5. Acute kidney injury until hospital discharge.
6. Severe intraventricular hemorrhage (IVH) until hospital discharge.
7. Cerebral palsy assessed at 18–24 months corrected age (CA), and at 3–5 years CA.
8. Severe developmental delay (Bayley Mental Developmental Index, or Griffiths Mental Development Scale), assessed as more than two standard deviations (SDs) below the mean at 18–24 months CA, and at 3–5 years CA.
9. Developmental delay (Bayley Mental Developmental Index, or Griffiths Mental Development Scale, reported as continuous outcome at 18–24 months CA, and at 3–5 years CA.
10. Severe intellectual impairment (intelligence quotient [IQ]), assessed as more than two SDs below the mean at 18–24 months CA, and at 3–5 years CA.
11. Intellectual impairment (intelligence quotient [IQ]), reported as continuous outcome at 18–24 months CA, and at 3–5 years CA.
12. Blindness (vision < 6/60 in both eyes).
13. Sensorineural deafness requiring amplification.

### Search methods

Search strategies were written and run by an Information Specialist (MF). Results were limited by methodological filters to identify randomized clinical trials and systematic reviews. Searches were conducted in October and November 2024 and run without language limits. Searches for trials were conducted without date limits; searches for conference abstracts in Embase were limited from 2021 forward; searches for systematic reviews were limited from 2023 forward. The following sources were searched:

- Ovid MEDLINE All, 1946 to 25 October 2024.
- Ovid Embase, 1974 to 25 October 2024.
- Cochrane Central Register of Controlled Trials, Issue 10, 2024, search date 25 October 2024.
- WHO ICTRP (International Clinical Trial Registry Platform), search date 08 November 2024.
- Clinicaltrials.gov, search date 08 November 2024.

We also conducted manual searches of the following conference abstracts: EAPS: European Academy of Paediatric Societies (2020–2023); PAS: Pediatric Academic Societies (2021–2024); and PSANZ: Perinatal Society of Australia and New Zealand (2020–2024).

Further details of the search, including full search strategies, are provided in the Supplementary Material.

### Data collection and analysis

We used the standard methods of Cochrane Neonatal, as indicated in the Supplementary Material.

For Summary of Finding (SoF) tables, we decided to use the following outcomes: all-cause mortality until hospital discharge; chronic lung disease assessed at 36 weeks’ postmenstrual age, defined as need for oxygen or respiratory support; any adverse event leading to caffeine cessation until hospital discharge, including tachycardia, dysrhythmia, feeding intolerance, irritability, and seizures; duration of mechanical ventilation until hospital discharge (days); duration of hospital stay until hospital discharge (days); apnea: number of infants with at least one episode (defined as interruption of breathing for more than 20 s) until hospital discharge; intermittent hypoxemia: number of infants with at least one episode until hospital discharge; acute kidney injury until hospital discharge; severe intraventricular hemorrhage until hospital discharge.

## Results

### Results of the search

**Fig. 1:**
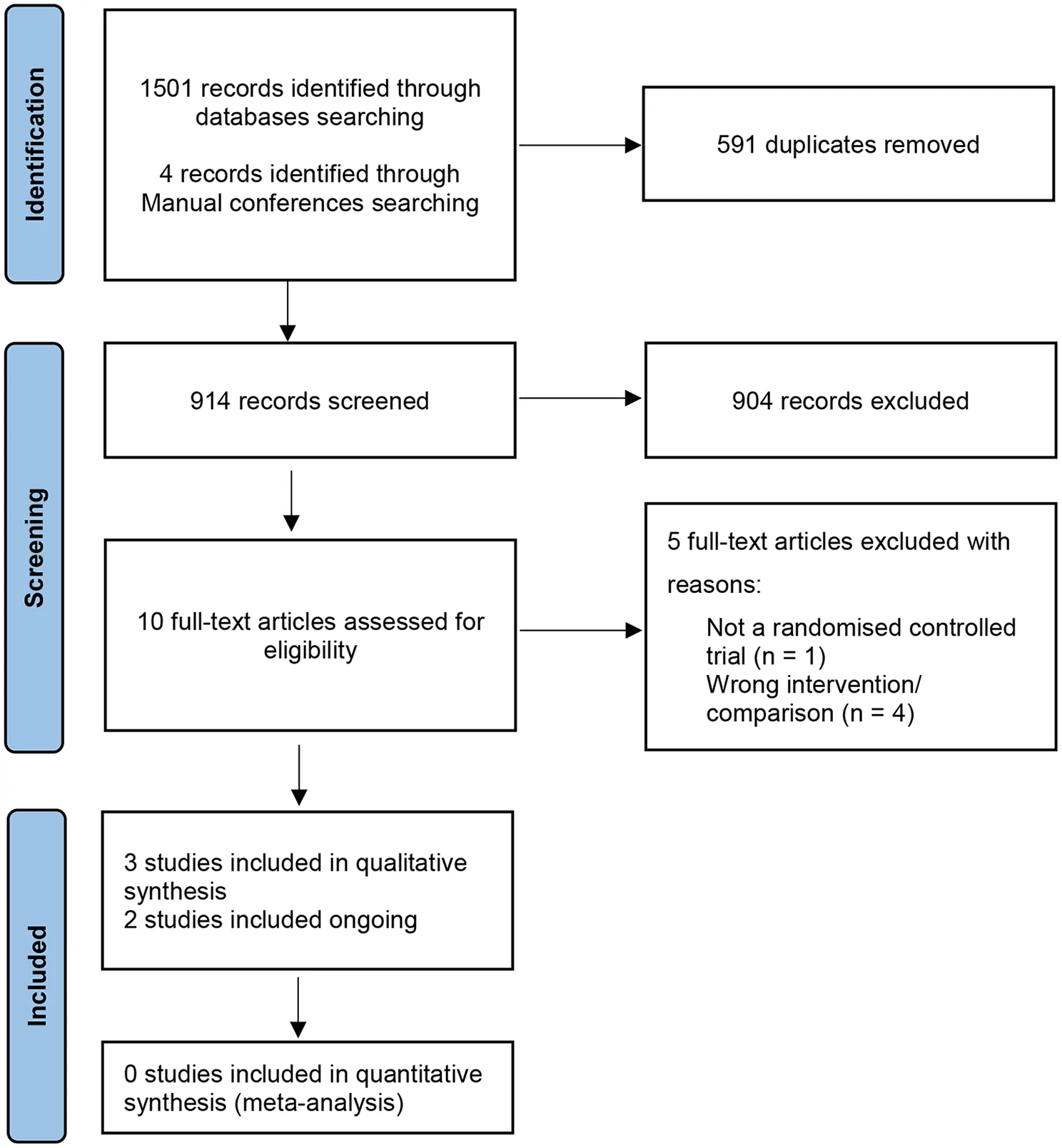
Study flow diagram

The search yielded 1507 references. After removing 601 duplicates, 906 records were available for screening. We excluded 890 based on titles/abstracts and assessed 16 full texts or trial registry records. We included 3 studies (6 references); excluded 6 studies (8 references); and identified 2 ongoing studies. The selection process is presented in Fig. 1.

### Description of studies

#### Included studies

We included three RCTs involving a total of 210 preterm infants: Dani 2023 [19]; Faramarzi 2018 [20]; Syed 2022 [21]. The latter did not report any outcomes relevant to this review, therefore the number of preterm infants included in the analysis is 78. An overview of included studies and Trials’ details are provided in Table 1 and the Supplementary Material, respectively. All included studies were single-center trials conducted in Italy (Dani 2023), Iran (Faramarzi 2018), and India (Syed 2022). Participants were preterm infants with a gestational age of 25–37 weeks. Sample sizes varied, ranging from 40 (Faramarzi 2018) to 128 (Syed 2022) infants. One study (Dani 2023) compared oral versus intravenous caffeine administration in the delivery room. An initial loading dose of 20 mg/kg caffeine citrate was administered intravenously or orally, followed by a maintenance dose of 5 mg/kg. Two studies (Faramarzi 2018, Syed 2022) compared twice-daily versus once-daily caffeine administration in preterm infants, both using a total daily dose of 5 mg/kg caffeine citrate.

**Table 1:** Characteristics of included studies

Study (design)	Country	Setting	Study period	Randomized (*n*)/ Sample size	Analyzed (*n*)	Intervention and comparator	Allocated (*n*)	GA (mean ± SD; range)	Birth weight (mean ± SD)
Dani 2023 (Parallel RCT)	Italy	One NICU	September 2019 – June 2021	42/38	38	Intervention: Caffeine citrate 20 mg/kg LD on day 1 within 10 min of birth, **enterally through orogastric tube**, then 5 mg /kg IV infusion MD every 24-hour	19	27.4 ± 1.2; 25–29	1003 ± 206
						Comparator: Caffeine citrate 20 mg/kg LD on day 1 within 10 min of birth, **IV via umbilical vein**, then 5 mg/kg IV infusion MD every 24-hour	19	27.9 ± 1.2; 25–29	983 ± 230
Faramarzi 2018 (Parallel RCT)	Iran	One NICU	July 2015 – August 2016	40/47	40	Intervention: Caffeine citrate 20 mg/kg IV LD on day 1, then **2.5 mg/kg** IV infusion MD over 20 min, **every 12-hour**	20	32.34 ± 3.46	1877 ± 742.5
						Comparator: Caffeine citrate 20 mg/kg LD on day 1, then **5 mg/kg** IV infusion MD over 20 min, **every 24-hour**	20	32.2 ± 3.06	1772 ± 675.27
Syed 2022 (Parallel RCT)	India	One Level IIIa NICU	June 2018 – June 2019	128/140	128	Intervention: Caffeine citrate 20 mg/kg LD, IV over 30 min, on day 1 of life, then **2.5 mg/kg** MD orally or IV infusion over 20 min, **every 12-hour**	64	30.94 ± 1.28; 28–33	2043.5 ± 181.13
						Comparator: Caffeine citrate 20 mg/kg LD, IV over 30 min, on day 1 of life, then **5 mg/kg** MD orally or IV infusion over 20 min, **every 24-hour**	64	31.14 ± 1.26; 29–33	1982.25 ± 181.13

Abbreviations: GA -gestational age; IV- intravenous; LD-loading dose; MD- maintenance dose; NICU-neonatal intensive care unit; RCT-randomised controlled trial

#### Excluded studies

During the full-text screening, we excluded six studies. Five studies were excluded because their interventions did not meet the review’s inclusion criteria: Katheria 2023 [22], Dobson 2017 [23], NCT00809055; NCT03340727; Srinivasan 2002 [24] and one was not an RCT (NCT06416956). Further details are available in the Supplementary Material.

#### Ongoing studies

We identified two ongoing studies: one comparing intravenous versus oral administration of the initial caffeine dose (CTRI/2023/07/055443 2023 [25]) and another evaluating caffeine dose adjustment as the infant grows versus maintaining a fixed dose over time (CTRI/2023/09/057903 2023 [26]). Further details are available in the Supplementary Material.

#### Risk of bias in included studies

Since the Syed 2022 study did not report any relevant outcomes, we were unable to assess its risk of bias. A detailed assessment of the risk of bias for all relevant outcomes in the Dani 2023 and Faramarzi 2018 studies is provided in the Supplementary Material. Faramarzi 2018 study did not include details about the randomization process, leading to its classification as having some concerns regarding bias arising from randomization. For all reported outcomes in the included studies, the risk of bias due to deviations from the intended interventions was also assessed as having some concerns. Although the participants were infants and therefore unaware of their assigned interventions, caregivers were aware of the treatment allocation. Other domains of risk of bias were assessed as low. As certainty of the evidence was downgraded three levels for extremely serious imprecision for all outcomes, further downgrade for risk of bias was not feasible.

### Synthesis of results

#### Is starting with an initial dose of intravenous caffeine better than starting with an oral dose?

Summary of effects (SoF1) is based on Dani 2023 study, which included 38 infants aged 25 ± 0–29 ± 6 weeks of gestation at high risk for respiratory distress syndrome (RDS), who do not require MV in the delivery room. Table 2 below shows summary of effects for the comparison intravenous versus oral administration. Very low certainty evidence for the following outcomes: all-cause mortality until hospital discharge (RR 0.14, 95% CI 0.01-2.59); CLD assessed at 36 weeks’ PMA (RR 5.00, 95%CI 0.26–97.70); duration of MV until hospital discharge (MD 1.00 days, 95% CI -8.05 to 10.05); duration of hospital stay until hospital discharge (MD days 8.00, 95% CI -5.35-21.35); severe intraventricular hemorrhage until hospital discharge (RR 1.00, 95%CI 0.29–3.43).

**Table 2:** Summary of findings table for the comparison intravenous versus oral administration

Summary of findings:						
Intravenous administration compared to oral administration in preterm infants						
**Patient or population**: preterm infants **Setting**: **Intervention**: Intravenous administration **Comparison**: oral administration						
Outcomes	Anticipated absolute effects^*^ (95% CI)		Relative effect (95% CI)	№ of participants (studies)	Certainty of the evidence (GRADE)	Comments
	**Risk with oral administration**	**Risk with Intravenous administration**				
All-cause mortality until hospital discharge	158 per 1000	**22 per 1000** (2 to 409)	**RR 0.14** (0.01 to 2.59)	38 (1 RCT)	⨁◯◯◯ Very low^a^	The evidence is very uncertain about the effect of intravenous administration of caffeine on all-cause mortality until hospital discharge compared to oral administration of caffeine in preterm infants.
Chronic lung disease (need for oxygen or respiratory support at 36 weeks’ postmenstrual age)	0 per 1000	**0 per 1000** (0 to 0)	**RR 5.00** (0.26 to 97.70)	38 (1 RCT)	⨁◯◯◯ Very low^a^	The evidence is very uncertain about the effect of intravenous administration of caffeine on CLD, defined as the need of oxygen or respiratory support at 36 weeks’ post menstrual age, compared to oral administration of caffeine.
Any adverse event leading to caffeine cessation	Not pooled	Not pooled	Not pooled	(0 studies)	-	
Duration of mechanical ventilation	The mean duration of mechanical ventilation was **0** day	MD **1 day higher** (8.05 lower to 10.05 higher)	-	38 (1 RCT)	⨁◯◯◯ Very low^a^	The evidence is very uncertain about the effect of intravenous administration of caffeine on the duration of mechanical ventilation slightly, compared to oral administration of caffeine.
Duration of hospital stay	The mean duration of hospital stay was **0** day	MD **8 day higher** (5.35 lower to 21.35 higher)	-	38 (1 RCT)	⨁◯◯◯ Very low^a^	The evidence is very uncertain about the effect of intravenous administration of caffeine on the duration of hospital stay, compared to oral administration of caffeine.
Apnea (interruption of breathing for more than 20 s)	Not pooled	Not pooled	Not pooled	(0 studies)	-	
Intermittent hypoxemia (IH)	Not pooled	Not pooled	Not pooled	(0 studies)	-	
Acute kidney injury until hospital discharge	Not pooled	Not pooled	Not pooled	(0 studies)	-	
Severe intraventricular hemorrhage until hospital discharge	211 per 1000	**211 per 1000** (61 to 722)	**RR 1.00** (0.29 to 3.43)	38 (1 RCT)	⨁◯◯◯ Very low^a^	The evidence is very uncertain about the effect of intravenous administration of caffeine on severe intraventricular hemorrhage, compared to oral administration of caffeine.

*The risk in the intervention group (and its 95% confidence interval) is based on the assumed risk in the comparison group and the **relative effect** of the intervention (and its 95% CI)

**CI**: confidence interval; **MD**: mean difference; **OR**: odds ratio; **RR**: risk ratio

**GRADE Working Group grades of evidence**

**High certainty**: we are very confident that the true effect lies close to that of the estimate of the effect

**Moderate certainty**: we are moderately confident in the effect estimate: the true effect is likely to be close to the estimate of the effect, but there is a possibility that it is substantially different

**Low certainty**: our confidence in the effect estimate is limited: the true effect may be substantially different from the estimate of the effect

**Very low certainty**: we have very little confidence in the effect estimate: the true effect is likely to be substantially different from the estimate of effect

*Explanations*

^a^Downgraded for extremly serious imprecision (three levels): one small studies, very wide CI

#### Is twice-daily (split dose) caffeine administration better than once-daily caffeine administration?

Summary of effects (SoF2) is based on Faramarzi 2018 study, which included 40 infants aged 26 ± 0–37 weeks of gestation with evidence of RDS that was diagnosed by a neonatologist and treated by caffeine therapy facilitating earlier weaning process from MV or undergoing continuous positive airway pressure (CPAP) therapy. Table 3 below shows summary of effects for the comparison twice versus once daily caffeine administration. Very low certainty evidence for the outcomes of all-cause mortality until hospital discharge (RR 1.00, 95%CI 0.29–3.45), duration of MV until hospital discharge (MD 0.81 days, 95% CI -7.56-9.18) and duration of hospital stay until hospital discharge (MD -2.70 days, 95% CI -8.12-2.72).

**Table 3:** Summary of findings table for the comparison twice versus once daily administration

Summary of findings:						
Twice-daily caffeine (split dose) compared to once-daily caffeine in preterm infants						
**Patient or population**: preterm infants **Setting**: **Intervention**: Twice-daily caffeine (split dose) **Comparison**: once-daily caffeine						
Outcomes	Anticipated absolute effects^*^ (95% CI)		Relative effect (95% CI)	№ of participants (studies)	Certainty of the evidence (GRADE)	Comments
	**Risk with once-daily caffeine**	**Risk with Twice-daily caffeine (split dose)**				
All-cause mortality until hospital discharge	200 per 1000	**200 per 1000** (58 to 690)	**RR 1.00** (0.29 to 3.45)	40 (1 RCT)	⨁◯◯◯ Very low^a^	The evidence is very uncertain about the effect of twice daily (split caffeine dose) on all cause mortality compared to once daily caffeine.
Chronic lung disease (need for oxygen or respiratory support at 36 weeks’ postmenstrual age)	Not pooled	Not pooled	Not pooled	(0 studies)	-	No studies reported on this outcome
Any adverse event leading to caffeine cessation	Not pooled	Not pooled	Not pooled	(0 studies)	-	No studies reported on this outcome
Duration of mechanical ventilation	The mean duration of mechanical ventilation was **0** days	MD **0.81 days higher** (7.56 lower to 9.18 higher)	-	40 (1 RCT)	⨁◯◯◯ Very low^a^	The evidence is very uncertain about the effect of twice daily (split caffeine dose) on the duration of mechanical ventilation compared to once daily caffeine.
Duration of hospital stay	The mean duration of hospital stay was **0** days	MD **2.7 days lower** (8.12 lower to 2.72 higher)	-	40 (1 RCT)	⨁◯◯◯ Very low^a^	The evidence is very uncertain about the effect of twice daily (split caffeine dose) o the duration of hospital stay compared to once daily caffeine.
Apnea (interruption of breathing for more than 20 s)	Not pooled	Not pooled	Not pooled	(0 studies)	-	No studies reported on this outcome
Intermittent hypoxemia (IH)	Not pooled	Not pooled	Not pooled	(0 studies)	-	No studies reported on this outcome
Acute kidney injury until hospital discharge	Not pooled	Not pooled	Not pooled	(0 studies)	-	No studies reported on this outcome
Severe intraventricular hemorrhage until hospital discharge	Not pooled	Not pooled	Not pooled	(0 studies)	-	No studies reported on this outcome

*The risk in the intervention group (and its 95% confidence interval) is based on the assumed risk in the comparison group and the **relative effect** of the intervention (and its 95% CI)

**CI**: confidence interval; **MD**: mean difference; **OR**: odds ratio; **RR**: risk ratio

**GRADE Working Group grades of evidence**

**High certainty**: we are very confident that the true effect lies close to that of the estimate of the effect

**Moderate certainty**: we are moderately confident in the effect estimate: the true effect is likely to be close to the estimate of the effect, but there is a possibility that it is substantially different

**Low certainty**: our confidence in the effect estimate is limited: the true effect may be substantially different from the estimate of the effect

**Very low certainty**: we have very little confidence in the effect estimate: the true effect is likely to be substantially different from the estimate of effect

*Explanations*

^a^Downgraded for extremly serious imprecision (three levels): one small studies, very wide CI

## Discussion

### Summary of main results

We evaluated the benefits and harms of different strategies for caffeine administration in preterm infants born at < 37 weeks’ gestation, at a PMA up to 44 weeks and 0 days, who receive caffeine for any indication. Our critical outcomes were all-cause mortality until hospital discharge, CLD assessed at 36 weeks’ PMA, and any adverse event leading to caffeine cessation until hospital discharge.

We included three RCTs totaling 210 preterm infants aged 25–37 weeks of gestation. One study compared intravenous to oral administration of the initial caffeine dose in the delivery room (Dani 2023). The evidence is very uncertain about the effect of intravenous administration of caffeine on all-cause mortality, severe IVH, duration of MV, duration of hospital stay, and CLD compared to the oral administration of caffeine.

Two studies compared twice-daily to once-daily caffeine administration (Faramarzi 2018 and Syed 2022). Our evidence is based on Faramarzi 2018 study, as Syed 2022 study did not report any of our outcomes of interest. The evidence is very uncertain about the effect of twice-daily caffeine administration on all-cause mortality, as well as, on the duration of MV and the duration of hospital stay, compared to once-daily caffeine administration.

No studies were included in the comparisons for the dose modification or adjunct (surfactant) therapy to caffeine. We identified two ongoing studies (CTRI/2023/07/055443 2023; CTRI/2023/09/057903 2023).

### Limitations of the evidence included in the review

All three included studies were RCTs, and their participants aligned with the predefined inclusion criteria. However, not all planned interventions or outcomes were addressed. No studies were identified that investigated dose modification strategies or adjunct therapy with surfactant. Additionally, none of the included studies reported adverse events leading to caffeine cessation, the short-term outcomes such as apnea, intermittent hypoxemia, or acute kidney injury, nor the predefined long-term neurodevelopmental outcomes. For all outcomes, the certainty of the evidence was downgraded to very low due to extremely serious imprecision (small studies). A few outcomes could have been downgraded for study limitations as well.

### Limitations of the review processes

This systematic review was conducted following Cochrane standard methods. None of the review authors were involved in any of the included studies. However, there is a possibility of intellectual bias, as some authors have conducted research on caffeine administration in preterm infants. The clinical questions addressed in this review have been identified following a priority exercise to develop national guidelines on the use of caffeine in preterm infants.

### Agreements and disagreements with other studies or reviews

Several Cochrane reviews have examined the benefits and risks of different caffeine dosing regimens and comparisons between caffeine and placebo or other drugs [6, 11, 27]. However, few retrospective studies and reviews have specifically addressed the interventions included in our review.

A retrospective cohort study assessing the effectiveness and safety of early (within two days of birth) versus late oral caffeine administration found improved outcomes with early treatment. These benefits included reduced duration of non-invasive mechanical ventilation, shorter hospital stays, faster achievement of full enteral feeding, and lower rates of BPD and osteopenia of prematurity [28]. A retrospective study found minimal benefit of twice-daily dosing in reducing the number of apneic episodes [29].

Some studies have highlighted the need for dose modifications. A previous review [30] suggested that increasing caffeine citrate by 1 mg/kg per day every 1–2 weeks until reaching 8 mg/kg per day may help maintain its therapeutic effect. While the American Academy of Pediatrics Committee on Fetus and Newborn does not recommend routine caffeine level monitoring, some infants may require plasma concentrations above 15 mg/L for optimal therapeutic benefit [31, 32]. Additionally, an age-adjusted dosing approach has been suggested to prevent caffeine levels from dropping below 15 mg/L after the second postnatal week with standard dosing or reaching potentially toxic levels in the first days of life with high-dose regimens. This recommendation is based on pharmacokinetic modeling and retrospective studies [33, 34]. The European Consensus Guidelines on the Management of RDS: 2022 Update suggest: “Gradual increase in dosing from 5 towards 8 mg/kg/day over several weeks may give the best chance of maintaining therapeutic effect” [35]. The NICE guidelines, in the presence of persistent apnea, suggest increasing the maintenance dosage (up to 20 mg/kg), splitting it into two doses, and increasing even further if needed under plasma level monitoring [36]. However, caution should be used to rely on this recommendation as it is based on a single, small RCT.

Finally, the effectiveness of combining caffeine with Less Invasive Surfactant Administration (LISA) was assessed in an RCT [22]. The study reported a lower incidence of endotracheal intubation within the first 72 h of life in the combination group. However, this study compared caffeine plus LISA with caffeine plus CPAP alone, which differs from the intervention of interest in our review. Current studies mainly aim to compare LISA vs. no LISA in the context of caffeine use, rather than comparing LISA + caffeine vs. LISA alone [22]. These questions need to be addressed in randomized controlled trials.

## Conclusions

This systematic review highlights that, while caffeine remains a cornerstone therapy for preterm infants, evidence on the optimal administration strategy—whether by route, frequency, or dose adjustment—is still very limited, and for some of these questions, evidence is completely lacking. No clear benefits could be established for intravenous versus oral administration or twice-daily versus once-daily dosing, and evidence on dose modifications and surfactant co-administration is currently absent.

These findings underscore an important gap in neonatal research. Given that caffeine is one of the most widely used drugs in neonatal intensive care, the questions addressed in this review are highly relevant to both clinical practice and future guideline development. Optimizing caffeine administration has the potential to improve survival, reduce complications such as chronic lung disease, and enhance neurodevelopmental outcomes in this highly vulnerable population. Well-designed, adequately powered randomized controlled trials are urgently needed to provide robust evidence. Data from the two ongoing studies may provide additional evidence on the effects of intravenous versus oral caffeine administration and the benefits of adjusting caffeine dosage as infants grow compared to maintaining a fixed dose over time.

## Supplementary Information

Below is the link to the electronic supplementary material.

Supplementary Material 1

## Data Availability

The following are made available: Search strategies; characteristics of included, excluded and ongoing studies; risk of bias assessments; and analyses. The data extraction form from Covidence is available from the authors on request.

## Abbreviations

GA: Gestational age
AOP: Apnea of prematurity
BPD: Bronchopulmonary dysplasia
GRADE: Grading of Recommendations, Assessment, Development, and Evaluation
PRISMA: Preferred Reporting Items for Systematic Reviews and Meta-Analyses
RCTs: Randomized controlled trials
PMA: Postmenstrual age
CLD: Chronic lung disease
MV: Mechanical ventilation
IVH: Intraventricular hemorrhage
CA: Corrected age
SDs: Standard deviations
IQ: Intelligence quotient
ICTRP: International Clinical Trial Registry Platform
EAPS: European Academy of Paediatric Societies
PAS: Pediatric Academic Societies
PSANZ: Perinatal Society of Australia and New Zealand
SoF: Summary of Findings
RDS: Respiratory distress syndrome
CPAP: Continuous positive airway pressure
NICU: Neonatal intensive care unit
LISA: Less Invasive Surfactant Administration
RoB 2.0: Risk of Bias tool, version 2.0
PROSPERO: International Prospective Register of Systematic Reviews
GA: Gestational age
AOP: Apnea of prematurity
BPD: Bronchopulmonary dysplasia
GRADE: Grading of Recommendations, Assessment, Development, and Evaluation
PRISMA: Preferred Reporting Items for Systematic Reviews and Meta-Analyses
RCTs: Randomized controlled trials
PMA: Postmenstrual age
CLD: Chronic lung disease
MV: Mechanical ventilation
IVH: Intraventricular hemorrhage
CA: Corrected age
SDs: Standard deviations
IQ: Intelligence quotient
ICTRP: International Clinical Trial Registry Platform
EAPS: European Academy of Paediatric Societies
PAS: Pediatric Academic Societies
PSANZ: Perinatal Society of Australia and New Zealand
SoF: Summary of Findings
RDS: Respiratory distress syndrome
CPAP: Continuous positive airway pressure
NICU: Neonatal intensive care unit
LISA: Less Invasive Surfactant Administration
