# Supplementary material for: Strategies for caffeine administration in preterm infants: route, dosing, monitoring, and adjunctive interventions - a systematic review
Source: Ital J Pediatr. 2026 Sep 21;52:143. doi: 10.1186/s13052-026-02344-0 (PMC13602543; doi:10.1186/s13052-026-02344-0)
Supplement: Supplementary file 1 [file 13052_2026_2344_MOESM1_ESM.docx]

# Supplementary material

*Strategies for caﬀeine administration in preterm infants- a systematic review*.

Alaa AM Osman, Chiara Russo, Maria Rojas-Reyes, Franciszek Borys, Michelle Fiander, Roger Soll, Matteo Bruschettini, Greta Sibrecht

**Table of Contents**

[***Supplementary material***](#_Toc198820505)

[Search strategies and methods](#_Toc198820506)

[Selection of studies](#_Toc198820507)

[Data extraction and management](#_Toc198820508)

[Risk of bias assessment in included studies](#_Toc198820509)

[Measures of treatment eﬀect](#_Toc198820510)

[Unit of analysis issues](#_Toc198820511)

[Dealing with missing data](#_Toc198820512)

[Reporting bias assessment](#_Toc198820513)

[Synthesis methods](#_Toc198820514)

[Investigation of heterogeneity and subgroup analysis](#_Toc198820515)

[Equity-related assessment](#_Toc198820516)

[Sensitivity analysis](#_Toc198820517)

[Certainty of the evidence assessment](#_Toc198820518)

[Consumer involvement](#_Toc198820519)

[**Results**](#_Toc198820520)

[Characteristics of included studies ordered by study ID](#_Toc198820521)

[References to included studies](#_Toc198820522)

[Characteristics of excluded studies (ordered by Study ID)](#_Toc198820523)

[References to excluded studies](#_Toc198820524)

[Characteristics of ongoing studies](#_Toc198820525)

[References to ongoing studies](#_Toc198820526)

[Risk of bias of included studies](#_Toc198820527)

[Analyses](#_Toc198820528)

[Figures: forest plots](#_Toc198820529)

### Search strategies and methods

Searches for trials were not limited by date; searches for systematic reviews were limited from 2023 forward; searches for conference abstracts in Embase were limited from 2021 forward.
The following databases were searched on 25 October 2024:

- Ovid MEDLINE(R) All, 1946 to Daily Update
- Ovid Embase, 1974-
- Cochrane CENTRAL via CRS

Two clinical trials registries were searched for ongoing or recently completed trials on 08 November 2024:

- US National Library of Medicine (<https://clinicaltrials.gov>)
- World Health Organization’s International Trials Registry Platform (<https://trialsearch.who.int/Default.aspx>)

**Other resources**

The following conference abstracts were searched:

- European Academy of Paediatric Societies (EAPS), 2020-2023
- Pediatric Academic Societies (PAS), 2021-2024
- Perinatal Society of Australia and New Zealand (PSANZ), 2021-2024

We reviewed the reference lists of included studies and related systematic reviews. We searched for errata or retractions for included studies in PubMed and the Retraction Watch Database.

##### Search narrative

The search strategy is comprised of terms for caffeine and the neonatal population.

The search logic is: Caffeine AND Neonates AND Methodological Filters.

#### *Summary of search results*

| Search summary |  |  |
| --- | --- | --- |
| October-25-2024 | Ovid MEDLINE(R) All | 347 |
| October-25-2024 | Embase 1974 to 2024 October 23 | 551 |
| October-25-2024 | Cochrane CRS | 479 |
| October-25-2024 | Conference abstracts | 6 |
| November-08-2024 | Trial registries | 124 |
|  | Total | 1507 |
|  | Duplicates | 601 |
|  | Net screened | 906 |

###### *MEDLINE*

|  | Ovid MEDLINE(R) All |  |
| --- | --- | --- |
|  | Search date: October-25-2024 |  |
| # | Searches | Results |
| 1 | Caffeine/ | 26034 |
| 2 | (caffeine or cafeine or caffein? or coffein? or guaranine or guarin or methyltheobromine? or trimethylxanthin$).ti,ab,kw,kf. | 34587 |
| 3 | (METHYLXANTHIN* or methyl* xanthin*).ti,ab,kw,kf. | 6415 |
| 4 | xanthines/ or xanthine/ | 8271 |
| 5 | (XANTHIN* or dioxopurin* or dioxypurin*).ti,ab,kw,kf. | 20220 |
| 6 | or/1-5 [Caffeine/Xanthines] | 66833 |
| 7 | exp Infant, Newborn/ or Intensive Care, Neonatal/ or Intensive Care Units, Neonatal/ or Gestational Age/ | 745608 |
| 8 | (babe or babes or baby* or babies or gestational age? or infant? or infantile or infancy or low birth weight or low birthweight or neonat* or neo-nat* or newborn* or new born? or newly born or premature or pre-mature or pre-matures or prematures or prematurity or pre-maturity or preterm or preterms or pre term? or preemie or preemies or premies or premie or VLBW or VLBWI or VLBW-I or VLBWs or LBW or LBWI or LBWs or ELBW or ELBWI or ELBWs or NICU or NICUs).ti,ab,kw,kf. | 1100336 |
| 9 | or/7-8 [Filter: Neonatal Population 04-2022-MEDLINE] | 1415476 |
| 10 | (clinical trial phase iii or controlled clinical trial or equivalence trial or pragmatic clinical trial or randomized controlled trial).pt. | 719987 |
| 11 | controlled clinical trials as topic/ or randomized controlled trials as topic/ or equivalence trials as topic/ or intention to treat analysis/ or pragmatic clinical trials as topic/ | 184170 |
| 12 | Random Allocation/ | 107734 |
| 13 | Double-Blind Method/ | 180952 |
| 14 | Single-Blind Method/ | 34138 |
| 15 | Placebos/ | 36015 |
| 16 | Control Groups/ | 2135 |
| 17 | (random* or sham or placebo*).ti,ab,hw,kf,kw. | 1959048 |
| 18 | ((singl* or doubl*) adj (blind* or dumm* or mask*)).ti,ab,hw,kf,kw. | 279979 |
| 19 | ((tripl* or trebl*) adj (blind* or dumm* or mask*)).ti,ab,hw,kf,kw. | 1946 |
| 20 | (control* adj3 (study or studies or trial* or group*)).ti,ab,kf,kw. | 1336135 |
| 21 | (quasi-random* or quasirandom*).ti,ab,hw,kf,kw. | 5882 |
| 22 | allocated.ti,ab,hw. | 91783 |
| 23 | ((open label or open-label) adj5 (study or studies or trial*)).ti,ab,hw,kf,kw. | 49458 |
| 24 | ((equivalence or superiority or non-inferiority or noninferiority) adj3 (study or studies or trial*)).ti,ab,hw,kf,kw. | 14103 |
| 25 | (pragmatic study or pragmatic studies).ti,ab,hw,kf,kw. | 678 |
| 26 | ((pragmatic or practical) adj3 trial*).ti,ab,hw,kf,kw. | 8839 |
| 27 | ((quasiexperimental or quasi-experimental) adj3 (study or studies or trial*)).ti,ab,hw,kf,kw. | 14561 |
| 28 | (phase adj3 (III or "3") adj3 (study or studies or trial*)).ti,hw,kf,kw. | 38007 |
| 29 | or/10-28 | 2789593 |
| 30 | exp Animals/ or exp Animal Experimentation/ or exp Models, Animal/ or exp Vertebrates/ | 27580127 |
| 31 | (mice or mouse or porcine or rat or rats or rodent?).ti. | 1554013 |
| 32 | exp Humans/ or exp Human Experimentation/ | 22309032 |
| 33 | (or/30-31) not 32 [Animals] | 5376595 |
| 34 | retrospective studies/ or historically controlled study/ or case reports/ or observational study/ | 3770597 |
| 35 | (retrospective or case study or case report? or observational study).ti. | 604484 |
| 36 | 29 not (or/33-35) [RCT Filter--based on https://searchfilters.cadth.ca/link/122] | 2243841 |
| 37 | meta-analysis/ or "systematic review"/ or network meta-analysis/ [/ finds same as .pt. syntax] | 366939 |
| 38 | ((systematic* adj3 (review* or overview*)) or (methodologic* adj3 (review* or overview*))).ti,ab,kf,kw. | 385852 |
| 39 | ("overview of reviews" or "review of reviews" or umbrella review? or scoping review?).ti. | 29867 |
| 40 | ((integrative adj3 (review* or overview*)) or (collaborative adj3 (review* or overview*)) or (pool* adj3 analy*)).ti,ab,kf,kw. | 44296 |
| 41 | (data synthes* or data extraction* or data abstraction*).ti,ab,kf,kw. | 47699 |
| 42 | (hand search* or handsearch*).ti,ab,kf,kw. | 11793 |
| 43 | (mantel haenszel or peto or der simonian or dersimonian or fixed effect* or latin square*).ti,ab,kf,kw. | 39721 |
| 44 | meta-analysis as topic/ or network meta-analysis/ | 31080 |
| 45 | (meta analy* or metanaly* or meta regression* or metaregression*).ti,ab,kf,kw. | 325477 |
| 46 | (medline or cochrane or pubmed or medlars or embase or cinahl).ab. | 395505 |
| 47 | (cochrane or systematic review?).jw. | 21274 |
| 48 | or/37-47 [SR filter-ML; based on https://searchfilters.cadth.ca/link/33] | 735524 |
| 49 | 6 and 9 [Caffeine/Xanthines and Neonate] | 3090 |
| 50 | (49 and 36) not 48 [RCT Results] | 313 |
| 51 | 49 and 48 and ("2023" or "2024").yr. [SR Results] | 34 |
| 52 | or/50-51 [Results MEDLINE] | 347 |

###### *Embase*

|  | Embase 1974 to 2024 October 23 |  |
| --- | --- | --- |
|  | Search date: October-25-2024 |  |
| # | Searches | Results |
| 1 | caffeine/ or xanthine derivative/ or xanthine/ or methylxanthine/ | 63355 |
| 2 | (caffeine or cafeine or caffein? or coffein? or guaranine or guarin or methyltheobromine? or trimethylxanthin$).ti,ab,kw,kf. | 41948 |
| 3 | (Methylxanthin* or methyl* xanthin*).ti,ab,kw,kf. | 7404 |
| 4 | (XANTHIN* or dioxopurin* or dioxypurin*).ti,ab,kw,kf. | 23091 |
| 5 | or/1-4 [Caffeine/Xanthine] | 92602 |
| 6 | newborn/ or prematurity/ or newborn intensive care/ or newborn care/ or gestational age/ | 840098 |
| 7 | (babe or babes or baby* or babies or gestational age? or infant? or infantile or infancy or low birth weight or low birthweight or neonat* or neo-nat* or newborn* or new born? or newly born or premature or pre-mature or pre-matures or prematures or prematurity or pre-maturity or preterm or preterms or pre term? or preemie or preemies or premies or premie or VLBW or VLBWI or VLBW-I or VLBWs or LBW or LBWI or LBWs or ELBW or ELBWI or ELBWs or NICU or NICUs).ti,ab,kw,kf. | 1326038 |
| 8 | or/6-7 [Filter: Neonatal Population 03-2022-OVID EMBASE] | 1609640 |
| 9 | randomized controlled trial/ or controlled clinical trial/ or equivalence trial/ or pragmatic trial/ | 1047275 |
| 10 | phase 3 clinical trial/ | 79000 |
| 11 | "randomized controlled trial (topic)"/ or "controlled clinical trial (topic)"/ | 293467 |
| 12 | randomization/ | 100243 |
| 13 | double blind procedure/ | 225105 |
| 14 | single blind procedure/ | 56807 |
| 15 | placebo/ | 419375 |
| 16 | control group/ | 110813 |
| 17 | (random* or sham or placebo*).ti,ab,hw,kf,kw. | 2750204 |
| 18 | ((singl* or doubl*) adj (blind* or dumm* or mask*)).ti,ab,hw,kf,kw. | 384362 |
| 19 | ((tripl* or trebl*) adj (blind* or dumm* or mask*)).ti,ab,hw,kf,kw. | 2536 |
| 20 | (control* adj3 (study or studies or trial* or group*)).ti,ab,kf,kw. | 1861471 |
| 21 | (quasi-random* or quasirandom*).ti,ab,hw,kf,kw. | 6761 |
| 22 | allocated.ti,ab,hw. | 118561 |
| 23 | ((open label or open-label) adj5 (study or studies or trial*)).ti,ab,hw,kf,kw. | 95606 |
| 24 | ((equivalence or superiority or non-inferiority or noninferiority) adj3 (study or studies or trial*)).ti,ab,hw,kf,kw. | 20936 |
| 25 | (pragmatic study or pragmatic studies).ti,ab,hw,kf,kw. | 1062 |
| 26 | ((pragmatic or practical) adj3 trial*).ti,ab,hw,kf,kw. | 10037 |
| 27 | ((quasiexperimental or quasi-experimental) adj3 (study or studies or trial*)).ti,ab,hw,kf,kw. | 22598 |
| 28 | (phase adj3 (III or "3") adj3 (study or studies or trial*)).ti,hw,kf,kw. | 141201 |
| 29 | or/9-28 [CADTH--NRCT terms removed https://searchfilters.cadth.ca/link/122 ] | 4036824 |
| 30 | exp animal/ or exp animal experiment/ or exp animal model/ or nonhuman/ or exp vertebrate/ | 34796746 |
| 31 | (mice or mouse or porcine or rat or rats or rodent?).ti. | 1744909 |
| 32 | exp human/ or exp human experiment/ | 27234779 |
| 33 | (or/30-31) not 32 [Animals] | 7591238 |
| 34 | 29 not 33 [RCT filter based on https://searchfilters.cadth.ca/link/122] | 3441124 |
| 35 | retrospective study/ | 1702185 |
| 36 | Cross-Over Studies/ or Crossover Procedure/ | 80083 |
| 37 | exp case control study/ | 244035 |
| 38 | case study/ or single-case study/ | 104400 |
| 39 | observational study/ | 398121 |
| 40 | (retrospective or case study or case report? or observational study).ti. | 763459 |
| 41 | or/35-40 [study designs excluded] | 2887757 |
| 42 | 34 not 41 [RCT filter FINAL excluding observational designs] | 2993450 |
| 43 | meta-analysis/ or "systematic review"/ or "meta analysis (topic)"/ [EMTREE] | 679949 |
| 44 | ((systematic* adj3 (review* or overview*)) or (methodologic* adj3 (review* or overview*))).ti,ab,kw,kf. | 466501 |
| 45 | ("overview of reviews" or "review of reviews" or umbrella review? or scoping review?).ti. | 30420 |
| 46 | ((integrative adj3 (review* or overview*)) or (collaborative adj3 (review* or overview*)) or (pool* adj3 analy*)).ti,ab,kw,kf. | 62125 |
| 47 | (data synthes* or data extraction* or data abstraction*).ti,ab,kw,kf. | 57976 |
| 48 | (hand search* or handsearch*).ti,ab,kw,kf. | 14392 |
| 49 | (mantel haenszel or peto or der simonian or dersimonian or fixed effect* or latin square*).ti,ab,kw,kf. | 52479 |
| 50 | (meta analy* or metanaly* or meta regression* or metaregression*).ti,ab,kw,kf. | 410193 |
| 51 | (medline or cochrane or pubmed or medlars or embase or cinahl).ab. | 495188 |
| 52 | (cochrane or systematic review?).jn,jx. | 33229 |
| 53 | or/43-52 [SR Filter: EMBASE based on CADTH filter: https://searchfilters.cadth.ca] | 1032614 |
| 54 | 5 and 8 [Caffeine/Xanthines and Neonate] | 5415 |
| 55 | (Conference Review or conference abstract).pt. | 5273461 |
| 56 | (54 and 42) not (or/53,55) [RCT Results not SRs or conference abstracts] | 490 |
| 57 | 54 and 42 and 55 and ("2021" or "2022" or "2023" or "2024").yr. [RCT Conference Abstracts 2021-2024] | 12 |
| 58 | 54 and 53 and ("2023" or "2024").yr. [SR Results 2023-2024] | 50 |
| 59 | or/56-58 [Results Embase] | 551 |

###### *Cochrane Central Register of Controlled Trials*

via Cochrane CRS (an internal Cochrane search interface)

|  | Cochrane CRS |  |
| --- | --- | --- |
|  | Search date: October-25-2024 |  |
| 1 | MESH DESCRIPTOR caffeine AND CENTRAL:TARGET | 2810 |
| 2 | (caffeine or cafeine or caffein* or coffein* or guaranine or guarin or methyltheobromine* or trimethylxanthin*):ti,ab,kw AND CENTRAL:TARGET | 5676 |
| 3 | (METHYLXANTHIN* or methyl* xanthin*):ti,ab,kw AND CENTRAL:TARGET | 339 |
| 4 | MESH DESCRIPTOR Xanthine AND CENTRAL:TARGET | 25 |
| 5 | MESH DESCRIPTOR Xanthines AND CENTRAL:TARGET | 183 |
| 6 | (XANTHIN* or dioxopurin* or dioxypurin*):ti,ab,kw AND CENTRAL:TARGET | 962 |
| 7 | #1 OR #2 OR #3 OR #4 OR #5 OR #6 | 7042 |
| 8 | MESH DESCRIPTOR Infant, Newborn EXPLODE ALL AND CENTRAL:TARGET | 23091 |
| 9 | MESH DESCRIPTOR Intensive Care, Neonatal AND CENTRAL:TARGET | 405 |
| 10 | MESH DESCRIPTOR Intensive Care Units, Neonatal AND CENTRAL:TARGET | 1253 |
| 11 | MESH DESCRIPTOR Gestational Age AND CENTRAL:TARGET | 5222 |
| 12 | ("babe" or "babes" or baby* or "babies" or "gestational age" or "gestational ages" or infant? or "infantile" or infancy or "low birth weight" OR "low birth weights" or "low birthweight" or "low birthweights" or neonat* or "neo-nat*" or newborn* or "new born?" or "newly born" or "premature" or "pre-mature" or "pre-matures" or prematures or prematurity or "pre-maturity" or "preterm" or "preterms" or "pre term?" or "preemie" or "preemies" or "premies" or "premie" or "VLBW" or "VLBWI" or "VLBW-I" or "VLBWs" or "LBW" or "LBWI" or "LBWs" or "ELBW" or "ELBWI" or "ELBWs" or "NICU" or "NICUs"):ti,ab,kw AND CENTRAL:TARGET | 91744 |
| 13 | #8 OR #9 OR #10 OR #11 OR #12 | 96412 |
| 14 | #7 AND #13 | 479 |

#### *Trial registries*

| Date | Site | Terms | Results |
| --- | --- | --- | --- |
| November-08-2024 | ICTRP | Homepage search: Caffeine and (neonate or infant or newborn or premature or prematurity) | 81 |
| November-08-2024 | ICTRP | Homepage search: Methylxanthine and (neonate or infant or newborn or premature or prematurity) | 4 |
| November-08-2024 | ctgov | Other terms: caffeine and (neonate or infant or newborn or premature or prematurity) \| Interventional studies | 32 |
| November-08-2024 | ctgov | Other terms: methylxanthine and (neonate or infant or newborn or premature or prematurity) \| Interventional | 7 |
|  |  | Total | 124 |

###### *Conferences*

| Conference Searches | | |
| --- | --- | --- |
| Process: Keyword search on website or within PDF of proceedings. Titles scanned and selected by MF. | | |
| Terms: |  |  |
| Conference | Results | Source |
| EAPS 2023 | 0 | Frontiers Events Abstracts. 10.3389/978-2-8325-1236-4 |
| EAPS 2022 | 0 | Frontiers Events Abstracts. 10.3389/978-2-88971-024-9 |
| EAPS 2021 | 0 | Frontiers Events Abstracts 10.3389/978-2-88966-546-4 |
| EAPS 2020 | 0 | Frontiers Events Abstracts 10.3389/978-2-88966-540-2 |
| PSANZ 2024 | 0 | Journal of Paediatrics and Child Health. 2024, 60(Suppl1) |
| PSANZ 2023 | 0 | Journal of Paediatrics and Child Health. 2023, 59(Suppl1) |
| PSANZ 2022 | 1 | Journal of Paediatrics and Child Health. 2022, 58(Suppl2) |
| PSANZ 2020 | 0 | Journal of Paediatrics and Child Health. 2020, 56(Suppl1) |
| PAS 2021 | 0 | <https://virtual2021.pas-meeting.org> |
| PAS 2022 | 0 | <https://2022.pas-meeting.org/> |
| PAS 2023 | 1 | <https://2023.pas-meeting.org> |
| PAS 2024 | 2 | <https://2024.pas-meeting.org> |
| Total | 4 |  |
| Key: EAPS: European Academy of Paediatric Societies; PAS: Pediatric Academic Societies; PSANZ: Perinatal Society of Australia and New Zealand | | |

### Selection of studies

Results of search were managed in Endnote [1]. The selection process was guided by the Criteria for considering studies for this review. Screening was conducted using Covidence [2].

Title/abstracts identiﬁed by literature searches were screened independently by two authors (MB, FB, or AO). Disagreements were resolved by discussion or in consultation with a third author (GS).

Full-texts of studies retained after the title/abstract screening were assessed independently by two authors (MB, ChR); disagreements were resolved by discussion or in consultation with a third author (GS).

We included studies reported as conference abstracts if they included suﬃcient data for extraction and analysis. In cases where there was insuﬃcient data, we contacted, or attempted to contact, study authors.

The selection process is reported both narratively and in a PRISMA ﬂow diagram [3].

### Data extraction and management

Two review authors (GS, AO, or ChR) independently extracted data. We piloted the form within the review team using a sample of two included studies. Data extracted by each review author were compared, and any disagreements resolved by discussion. Data was entered into RevMan [4]. We extracted the following information:

- Administrative details: study author(s), published or unpublished, year of publication, year in which study was conducted, presence of vested interest, source of funding.
- Study setting: number of study centers and location, informed consent, ethics approval, completeness of follow-up (e.g. greater than 80%).
- Participants: number randomized, number lost to follow-up/withdrawn, number analyzed, mean GA, GA range, mean CA or CA range, inclusion criteria and exclusion criteria.
- Type of intervention, according to Types of interventions
- Outcomes, as outlined in Outcome measures. If we identify ongoing studies, we will document available information such as the primary author, research question(s), methods, and outcome measures, and the estimated date of completion in a 'Characteristics of ongoing studies' table.

We resolved any disagreements by discussion. Two review authors (GS, MXR) entered data into RevMan [4].

### Risk of bias assessment in included studies

We used Cochrane RoB 2 Excel tool [5] to assess risk of bias in randomized trials and followed guidance from the Cochrane Handbook for Systematic Reviews of Interventions [6].

The outcomes to be assessed for each study are those described in Certainty of the evidence assessment.

Two review authors (AO, FB) independently assessed the risk of bias (low, high, or some concerns) for each critical outcome. Any discrepancies in judgment were resolved through discussion or by consultation with a third review author (GS).

- Bias arising from the randomization process
- Bias due to deviations from intended interventions (we will assess the eﬀect of assignment to the intervention at baseline, i.e. the 'intention-to-treat eﬀect')
- Bias due to missing outcome data
- Bias in measurement of the outcome Bias in selection of the reported result

To address these types of bias, we used the signaling questions recommended in RoB2 and made a judgment as follows:

- 'Yes': if there is ﬁrm evidence that the question [7] was fulﬁlled in the study (i.e. the study was at low or high risk of bias given the direction of the question).
- 'Probably yes': a judgment was made that the question was fulﬁlled in the study (i.e. the study was at low or high risk of bias given the direction of the question).
- 'No': if there was ﬁrm evidence that the question was unfulﬁlled in the study (i.e. the study was at low or high risk of bias given the direction of the question).
- 'Probably no': a judgment was made that the question was unfulﬁlled in the study (i.e. the study was at low or high risk of bias given the direction of the question).
- 'No information': if the study report provided insuﬃcient information to allow any judgment.

We used the algorithms proposed by RoB2 to assign each domain one of the following levels of bias:

- low risk of bias
- some concerns
- high risk of bias

This approach allowed the derivation an overall risk of bias rating for each outcome in each study as follows:

- 'Low risk of bias': we judged the trial at low risk of bias for all domains for this result.
- 'Some concerns': we judged the trial to raise some concerns in at least one domain for this result, but not as at high risk of bias for any domain.
- 'High risk of bias': we judged the trial to be at high risk of bias in at least one domain for the result, or we judged the trial to have some concerns for multiple domains such that our conﬁdence in the results is substantially lowered.

### Measures of treatment eﬀect

**Dichotomous data**

For dichotomous data we used risk ratio and risk diﬀerence, and 95% conﬁdence intervals (CIs). In instances where there would be a statistically signiﬁcant decrease (or increase) in risk diﬀerence, we computed the number needed to treat for an additional beneﬁcial outcome or the number needed to treat for an additional harmful outcome, also with 95% CIs.

**Continuous data**

For continuous data we used the mean diﬀerence for outcomes measured consistently across trials. For studies that measured the same outcome but employed diﬀerent methodologies, we would employ the standardized mean diﬀerence to consolidate the data. In cases where trial data were reported as median and interquartile range and passed the skewness test, we would convert medians to means and estimate the standard deviation as interquartile range divided by 1.35.

### Unit of analysis issues

The unit of analysis in RCTs is the individual neonate. An infant was only be considered once for analysis in RCTs. The unit of analysis in cluster‐randomized trials would be the participating neonatal unit or section of a neonatal unit or hospital. For cluster‐randomized trials, we would abstract information on the study design and unit of analysis for each study, indicating whether clustering of observations is present due to allocation to the intervention at the group level, or clustering of individually randomized observations (e.g. infants within clinics). We would abstract available statistical information needed to account for the implications of clustering on the estimation of outcome variances, such as design eﬀects or intracluster correlations (ICCs), and whether the study adjusted results for the correlations in the data. In cases where studies did not account for clustering, we would ensure that appropriate adjustments are made to the eﬀective sample size following Cochrane guidance. Where possible, we would derive the ICC for these adjustments from the trial itself, or from a similar trial. If an appropriate ICC is unavailable, we would conduct sensitivity analyses to investigate the potential eﬀect of clustering, by imputing a range of values of ICC.

We did not include cross‐over trials, as they do not allow the use of outcomes that require time to develop/discover.

If a trial had multiple arms compared to the same control condition, we would either combine groups to create a single pair-wise comparison, or select the pair of interventions that most closely matches the deﬁnitions given and exclude the others. We would include the arm where diﬀerent caﬀeine administration strategies are used compared to the control group where no speciﬁc intervention was introduced. If there were several arms with strategies, we would combine them into one group. We would acknowledge this potential selective bias of data used for analysis in the Discussion section of the review.

### Dealing with missing data

We intended to carry out analysis on an intention-to-treat basis for all included outcomes. Whenever possible, we analyzed all participants in the treatment group to which they were randomized, regardless of the actual treatment received. If we identiﬁed important missing data (in the outcomes) or unclear data, we would contact the original investigators and request the missing data. We would make explicit the assumptions of any methods used to deal with missing data.

For missing dichotomous outcomes, we would include participants with incomplete or missing data in the sensitivity analyzes by imputing them according to the following scenarios.

- Extreme‐case analysis favoring the experimental intervention (best‐worst case scenario): none of the dropouts/participants lost from the experimental arm, but all the dropouts/participants lost from the control arm experienced the outcome, including all randomized participants in the denominator
- Extreme‐case analysis favoring the control (worst‐best case scenario): all dropouts/participants lost from the experimental arm, but none from the control arm experienced the outcome, including all randomized participants in the denominator

For continuous outcomes, we would calculate missing standard deviations using reported P values or CIs. If calculation is not possible, we would impute a standard deviation as the highest standard deviation reported in the other trials for the corresponding treatment group and outcome.

### Reporting bias assessment

To assess reporting bias, we compared the predeﬁned primary and secondary outcomes to the reporting outcomes. In instances where study protocols were available, we compared them to the full publications to gauge the potential presence of reporting bias. Study involving interventions for a potentially eligible infant population but not reporting on any primary or secondary outcomes, is documented in the characteristics of included studies tables.

To screen for publication bias, we planned to employ funnel plots if a substantial number of studies (> 10) report the same outcome. However, our review comprises fewer than 10 studies suitable for meta-analysis, our ability to detect publication bias was limited.

### Synthesis methods

Given the number of studies identified for each comparison, meta-analysis was not possible. We were also unable to follow Synthesis Without Meta‐analysis (SWiM) reporting guidance per the Cochrane Handbook. We therefore used forest plots to graphically represent study data.

### Investigation of heterogeneity and subgroup analysis

We described the clinical diversity and methodological variability of the evidence narratively and in tables. Tables include data on study characteristics, such as design features, population characteristics, and intervention details.

We included too few studies to conduct subgroup analyses, but describe the methods we would have followed below.

We planned to carry out the following subgroup analyses of factors that may have contributed to heterogeneity in the eﬀects of the intervention.

- Gestational age: extremely preterm (less than 28 weeks); very preterm (less than 32 weeks); 32 weeks or more
- Dose: high dose; standard dose, using the deﬁnitions provided in the Cochrane review on caﬀeine dosing regimens

We planned to use the main outcomes (those speciﬁed for the summary of ﬁndings table) in subgroup analyses if there were enough studies reporting the outcomes to support valid subgroup comparisons (at least ﬁve studies per subgroup).

### Equity-related assessment

We did not investigate health inequity in this review due to limited resources.

### Sensitivity analysis

We were unable to conduct sensitivity analysis, but below find the planned methods had we included a sufficient number of studies.

We planned to conduct sensitivity analyses to explore the eﬀect of the methodological quality of studies, and to ascertain whether studies with a high risk of bias (in at least two domains) overestimate the eﬀect of treatment.

Diﬀerences in the study design of studies included in this review might also aﬀect our results. We planned to perform a sensitivity analysis to compare the eﬀects of higher initial doses of surfactant in truly randomized trials as opposed to quasi-randomized trials.

For missing dichotomous outcomes, we would include participants with incomplete or missing data in the sensitivity analyzes by imputing them according to best‐worst case scenario and worst‐best case scenario (see Dealing with missing data).

### Certainty of the evidence assessment

We used the GRADE approach, as outlined in the GRADE Handbook, to assess the certainty of evidence for the following outcomes [9].

- All-cause mortality until hospital discharge
- Chronic lung disease assessed at 36 weeks' postmenstrual age, deﬁned as need for oxygen or respiratory support
- Any adverse event leading to caﬀeine cessation until hospital discharge, including tachycardia, dysrhythmia, feeding intolerance, irritability, and seizures
- Duration of mechanical ventilation until hospital discharge (days) Duration of hospital stay until hospital discharge (days)
- Apnea: number of infants with at least one episode (deﬁned as interruption of breathing for more than 20 seconds) until hospital discharge
- Intermittent hypoxemia (IH): number of infants with at least one episode until hospital discharge Acute kidney injury until hospital discharge
- Severe intraventricular hemorrhage until hospital discharge

We prepared a summary of ﬁndings table for each comparison including at least one study.

Two review authors (MXR; MB) independently assessed the certainty of the evidence for each of the outcomes above. At any point in the assessment process, disagreements between review authors were resolved by discussion or by consultation with a third review author (GS). We considered evidence from randomized controlled trials as high certainty, downgrading the evidence one level for serious (or two levels for very serious) limitations based upon the following: design (overall risk of bias), consistency across studies, directness of the evidence, precision of estimates (up to three levels for extremely serious), and presence of publication bias. We used GRADEpro software [10] to create a summary of ﬁndings table to report the certainty of the evidence.

The GRADE approach results in an assessment of the certainty of a body of evidence in one of the following four grades.

- High: we are very conﬁdent that the true eﬀect lies close to that of the estimate of the eﬀect
- Moderate: we are moderately conﬁdent in the eﬀect estimate; the true eﬀect is likely to be close to the estimate of the eﬀect, but there is a possibility that it is substantially diﬀerent
- Low: our conﬁdence in the eﬀect estimate is limited; the true eﬀect may be substantially diﬀerent from the estimate of the eﬀect
- Very low: we have very little conﬁdence in the eﬀect estimate; the true eﬀect is likely to be substantially diﬀerent from the estimate of eﬀect

### Consumer involvement

We did not involve consumers in this review due to limited resources, although we used core outcome sets for the review's outcomes, which have been developed with consumer involvement. However, consumers were involved in the prioritization of the questions addressed in this review.

##### References

1	The EndNote Team. EndNote. Philadelphia, PA, USA: Clarivate; 2013.

2	Covidence. Melbourne, Australia: Veritas Health Innovation;

3	Page MJ, McKenzie JE, Bossuyt PM, Boutron I, Hoffmann TC, Mulrow CD. The PRISMA 2020 statement: an updated guideline for reporting systematic reviews. BMJ. 2021;372:n71.

4	Review Manager (RevMan). The Cochrane Collaboration

5	Sterne JA, Savović J, Page MJ, Elbers RG, Blencowe NS, Boutron I. RoB 2: a revised tool for assessing risk of bias in randomised trials. BMJ. 2019;366:l4898.

6	Higgins JP, Savović J, Page MJ, Elbers RG, Sterne JA. Chapter 8: Assessing risk of bias in a randomized trial. In: Higgins JP, Thomas J, Chandler J, Cumpston M, Li T, Page MJ, Welch VA editor(s). Cochrane Handbook for Systematic Reviews of Interventions Version 6.5 (updated August 2024). Cochrane, 2024. Available from [www.training.cochrane.org/handbook](http://www.training.cochrane.org/handbook).

7	Higgins JP, Eldridge S, Li T editor(s). Chapter 23: Including variants on randomized trials. In: Higgins JP, Thomas J, Chandler J, Cumpston M, Li T, Page MJ, Welch VA editor(s). Cochrane Handbook for Systematic Reviews of Interventions Version 6.4 (updated August 2023). Cochrane, 2023. Available from [www.training.cochrane.org/handbook](http://www.training.cochrane.org/handbook).

8	Higgins JP, Thomas J, Chandler J, Cumpston M, Li T, Page MJ, et al. Cochrane Handbook for Systematic Reviews of Interventions Version 6.4 (updated August 2023). Cochrane, 2023. Available from [www.training.cochrane.org/handbook](http://www.training.cochrane.org/handbook).

9	Schünemann H, Brożek J, Guyatt G, Oxman A, editor(s). Handbook for grading the quality of evidence and the strength of recommendations using the GRADE approach (updated October 2013). GRADE Working Group, 2013. Available from <https://gdt.gradepro.org/app/handbook/handbook.html>.

10	GRADEpro GDT. Hamilton (ON): McMaster University (developed by Evidence Prime).

## Results

### Characteristics of included studies ordered by study ID

| Dani 2023 | |
| --- | --- |
| Methods | Study design: Randomized controlled trial Study grouping: Parallel group |
| Participants | Intervention Gestational age(mean SD)/GA range: 27.4 ± 1.2 /25-29 Birth weight (mean SD): 1003 ± 206 Control Gestational age(mean SD)/GA range: 27.9 ± 1.2 /25-29 Birth weight (mean SD): 983 ± 230 Overall Gestational age (mean SD)/GA range: 25-29  Inclusion criteria: Infants aged 25±0–29±6 weeks of gestational age at high risk of developing RDS, who do not require mechanical ventilation in the delivery room. Exclusion criteria: Maternal consumption of caffeine before giving birth (> 2 cups of coffee in the 6-h prior birth) major congenital malformations and chromosomal syndromes. Group differences: Clinical characteristics were similar between the two groups Number randomized/Total sample size: 42/38 Number analyzed: 38 |
| Interventions | Intervention Number of participants allocated: 19 Dose: 20 mg/kg caffeine citrate within 10 min of birth followed by an IV maintenance dose of 5 mg/kg per day Route of administration: Enterally through an orogastric tube Frequency of caffeine administration (once or twice daily): Once daily co-intervention (surfactant or other respiratory drugs): Surfactant (Curosurf®, Chiesi, Parma, Italy) at 200 mg/kg Control Number of participants allocated: 19 Dose: 20 mg/kg caffeine citrate within 10 min of birth followed by an IV maintenance dose of 5 mg/kg per day Route of administration: Intravenous via the umbilical vein Frequency of caffeine administration (once or twice daily): Once daily co-intervention (surfactant or other respiratory drugs): Surfactant (Curosurf®, Chiesi, Parma, Italy) at 200 mg/kg |
| Outcomes | Trial’s primary outcome(s): Feasibility of IV or enteral caffeine administration in the DR during stabilization  Trial’s secondary outcome(s): The need for MV in treated infants in comparison with a historical matched control group  Reported outcomes considered for this review:  All-cause mortality until hospital discharge Outcome type: DichotomousOutcome Reported as: Number of participants with event (n, N) Unit of measure: rate (%) Direction: Lower is better Data value: Endpoint Duration of hospital stay Outcome type: ContinuousOutcome Reported as: Standard deviation (mean, SD, N) Unit of measure: days, mean +- SD Direction: Lower is better Data value: Endpoint Severe intraventricular hemorrhage (grade III and IV) Outcome type: DichotomousOutcome Reported as: Number of participants with event (n, N) Unit of measure: rate (%) Direction: Lower is better Data value: Endpoint Chronic lung disease CLD/BPD Outcome type: DichotomousOutcome Reported as: Number of participants with event (n, N) Unit of measure: rate (%) Direction: Lower is better Data value: Endpoint Notes: I'm not sure of the time of evaluation Duration of mechanical ventilation Outcome type: ContinuousOutcome Reported as: Standard deviation (mean, SD, N) Unit of measure: days, mean +- SD Direction: Lower is better Data value: Endpoint Notes: PTV duration |
| Identification | Sponsorship source: Open access funding provided by Università degli Studi di Firenze within the CRUI-CARE Agreement. The study was partially funded by a liberal contribute from Chiesi Farmaceutici Spa. Country: Italy Setting: Neonatal intensive care unit Comments: The study reported Duration of mechanical ventilation during hospitalization (days) categorized into PTV duration (day) or HFOV duration (day); I think this fit into our outcome of Duration of mechanical ventilation until hospital discharge (days), but we should report only the PTV duration because it is the longest duration, and we cannot combine the mean and SD of the two categories together. Author's name: Carlo Dani Institution: Division of Neonatology, Careggi University Hospital of Florence, Largo Brambilla 3, 50134, Florence, Italy Email: cdani@unifi.it Address: Division of Neonatology, Careggi University Hospital of Florence, Largo Brambilla 3, Florence, 50134, Italy Start date: September 2019 End date: June 2021 Number of study centers and locations: One; the NICU of the Careggi University Hospital of Florence Statements of interest: CD received honoraria from Chiesi Farmaceutici Spa and Vyaire Medical Inc. for scientific consultancy. Remaining authors declare that they have no competing interests. Length of follow-up: Until hospital discharge Informed consent: Parental consent was obtained prior to delivery. Ethics approval: Local ethics committees approved the study. Completeness of follow-up: Yes  Registration: NCT04044976 |
| Faramarzi 2018 | |
| Methods | Study design: Randomized controlled trial Study grouping: Parallel group |
| Participants | Intervention Gestational age(mean SD)/GA range: 32.34±3.46 Birth weight (mean SD): 1877±742.5 Control Gestational age(mean SD)/GA range: 32.2±3.06 Birth weight (mean SD): 1772±675.27 Overall Gestational age(mean SD)/GA range: 32.27±3.23 Birth weight (mean SD): 1824.5±702.54  Inclusion criteria: Gestational age of 26 to 37 weeks, with evidence of respiratory distress syndrome that was diagnosed by a neonatologist and treated by caffeine therapy facilitating earlier weaning process from mechanical ventilation or undergoing CPAP therapy. Exclusion criteria: Asphyxia, hypoglycemia, intracranial ventricular hemorrhage (IVH), major congenital anomaly and previous exposure to methylxanthine therapy. Group differences: No significant differences Number randomized/Total sample size: 40/47 Number analyzed: 40 |
| Interventions | Intervention Number of participants allocated: 20 Dose: 2.5 mg/kg infused every 12-hour-interval over 20 minutes Route of administration: Intravenous infusion Frequency of caffeine administration (once or twice daily): Twice daily co-intervention (surfactant or other respiratory drugs): Surfactant therapy (INSURE) Control Number of participants allocated: 20 Dose: 5mg/kg infused every 24-hour-interval over 20 minutes Route of administration: Intravenous infusion Frequency of caffeine administration (once or twice daily): Once daily co-intervention (surfactant or other respiratory drugs): Surfactant therapy (INSURE) |
| Outcomes | Trial’s primary outcome(s): CPAP failure and extubation failure during the treatment  Trial’s secondary outcome(s): Adverse reactions during the treatment  Reported outcomes considered for this review:  Duration of mechanical ventilation Outcome type: Continuous outcome Reported as: Standard deviation (mean, SD, N) Scale: mean±SD Unit of measure: days Direction: Lower is better Data value: Endpoint Duration of hospital stay Outcome type: Continuous outcome Reported as: Standard deviation (mean, SD, N) Scale: mean±SD Unit of measure: days Direction: Lower is better Data value: Endpoint All-cause mortality until hospital discharge Outcome type: DichotomousOutcome Reported as: Number of participants with event (n, N) Scale: n Direction: Lower is better Data value: Endpoint Any adverse event leading to caffeine cessation Outcome type: DichotomousOutcome Reported as: Number of participants with event (n, N) Data value: Endpoint |
| Identification | Sponsorship source: Mazandaran University of Medical Sciences Country: Iran Setting: Neonatal intensive care unit in Bu-Ali Sina Teaching Hospital, Sari, Iran. Author's name: Mohammadreza Rafati Institution: Department of Clinical Pharmacy, Faculty of pharmacy, Mazandaran University of Medical Sciences, Sari, Iran Email: mrrafati@mazums.ac.ir Address: Department of Clinical Pharmacy, Faculty of pharmacy, Mazandaran University of Medical Sciences, Sari, PO BOX: 48471-93698, Iran Start date: July 2015 End date: August 2016 Number of study centers and locations: One study center, neonatal intensive care unit in Bu-Ali Sina Teaching Hospital, Sari, Iran Statements of interest: No conflict of interest Informed consent: Informed signed consent was obtained from the parents of the patients. Ethics approval: Study protocol was approved by the Research Ethics Committee of Mazandaran University of Medical Sciences, Sari, Iran. Completeness of follow-up: 100%  Registration: IRCT201510172342N4 |

| **Syed 2022** | |
| --- | --- |
| Methods | Study design: Randomized controlled trial Study grouping: Parallel group |
| Participants | Intervention Gestational age(mean SD)/GA range: (30.94±1.28)/28-33 Birth weight (mean SD): 2043.50 ± 181.13 Control Gestational age(mean SD)/GA range: (31.14±1.26)/29-33 Birth weight (mean SD): 1982.25 ± 181.13  Inclusion criteria: Preterm neonates between 28 to 34 weeks of gestation with AOP defined as a pause breathing for more than 15 to 20 seconds, or accompanied by oxygen desaturation (SpO2 ≥ 80% for ≥ four seconds) and bradycardia (Heart rate < 2 / 3 of baseline for ≥ four seconds) Exclusion criteria: Neonates with birth asphyxia, meconium aspiration syndrome (MAS), hypoglycemia, intracranial ventricular haemorrhage (IVH), sepsis, haemodynamically significant cardiac anomaly, other major congenital anomalies and previous exposure to methylxanthine therapy. Group differences: None Number randomized/Total sample size: 128/140 Number analyzed: 128 |
| Interventions | Intervention Number of participants allocated: 64 Dose: 2.5 mg / kg of caffeine citrate (1.25 mg / kg as caffeine base) every 12-hour-interval Route of administration: Orally or IV infusion over 20 minutes Frequency of caffeine administration (once or twice daily): Twice daily co-intervention (surfactant or other respiratory drugs): Surfactant: intubate surfactant extubate (INSURE) or less invasive surfactant administration (LISA) as needed Control Number of participants allocated: 64 Dose: 5mg / kg of caffeine citrate (2,5 mg / kg as caffeine base) every 24-hour-interval Route of administration: orally or IV infusion over 20 minutes Frequency of caffeine administration (once or twice daily): Once daily co-intervention (surfactant or other respiratory drugs): Surfactant: intubate surfactant extubate (INSURE) or less invasive surfactant administration (LISA) as needed |
| Outcomes | Trial’s primary outcome(s): Number of apneic episodes and feed tolerance  Trial’s secondary outcome(s): Heart rate, daily weight and adverse effects  Reported outcomes considered for this review:  Apnea: number of infants with at least one episode Outcome type: DichotomousOutcome Reported as: Number of participants with event (n, N) Data value: Endpoint Any adverse event leading to caffeine cessation Outcome type: DichotomousOutcome Reported as: Number of participants with event (n, N) Data value: Endpoint |
| Identification | Sponsorship source: None Country: India Setting: Level IIIa neonatal intensive care unit Author's name: Sandesh Kini Institution: Department of Paediatrics, Kasturba Medical College, Manipal, Manipal Academy of Higher Education, Manipal, Karnataka, India Email: sandesh.kini@manipal.edu Address: Department of Paediatrics, Kasturba Medical College, Manipal, Manipal Academy of Higher Education, Manipal, Karnataka, India Start date: June 2018 End date: June 2019 Number of study centers and locations: One study centre, CRAFT Hospital and Research Centre, Thrissur, India. Statements of interest: None Length of follow-up: From the time of caffeine therapy initiation until the completion of treatment (until infants reached an age of 37 weeks or had five to seven days without significant apnea events) Informed consent: Informed signed consent was obtained from the parents of newborns who were recruited into the study. Ethics approval: Study protocol was approved by the institutional ethics committee. Completeness of follow-up: 100%  Registration: not found |

### References to included studies

**Dani 2023**

- Dani C, Cecchi A, Ciarcia M, Miselli F, Luzzati M, Remaschi G, Bona MD, la Marca G, Boni L. Enteral and Parenteral Treatment with Caffeine for Preterm Infants in the Delivery Room: A Randomised Trial. Paediatr Drugs 2023;25(1):79-86. [DOI: [https://dx.doi.org/10.1007/s40272-022-00541-y](https://doi.org/https:/dx.doi.org/10.1007/s40272-022-00541-y)]
- Dani C, Cecchi A, Remaschi G, Mercadante D, la Marca G, Boni L, Mosca F. Study protocol: treatment with caffeine of the very preterm infant in the delivery room: a feasibility study. BMJ Open 2020;10(12):e040105-. [DOI: [https://dx.doi.org/10.1136/bmjopen-2020-040105](https://doi.org/https:/dx.doi.org/10.1136/bmjopen-2020-040105)]
- NCT04044976. Treatment With Caffeine of Very Preterm Infant in the Delivery Room: the CAFROOM Feasibility Study. <https://clinicaltrials.gov/study/NCT04044976 2019>.

**Faramarzi 2018**

- Faramarzi F, Shiran M, Rafati M, Farhadi R, Salehifar E, Nakhshab M. The efficacy and safety of two different doses of caffeine in respiratory function of preterm infants. Caspian 2018;9(1):46-53. [DOI: [https://dx.doi.org/10.22088/cjim.9.1.46](https://doi.org/https:/dx.doi.org/10.22088/cjim.9.1.46)]
- IRCT201510172342N4. Evaluation and comparison of efficacy and adverse drug reactions for single or two divided caffeine dosing to treat apnea of prematurity in preterm neonates.
- https://trialsearch.who.int/Trial2.aspx?TrialID=IRCT201510172342N4 2017.

**Syed 2022**

- Syed F, Kini S, Majeed A. The Efficacy and Safety of Once Daily versus Twice Daily Dosing of Caffeine Citrate in Apnea of prematurity: a Randomised Control Trial. Journal of Nepal Paediatric Society 2022;42(1):124-128. [DOI: [https://dx.doi.org/10.3126/jnps.v42i1.37173](https://doi.org/https:/dx.doi.org/10.3126/jnps.v42i1.37173)]

### Characteristics of excluded studies (ordered by Study ID)

| Study | Reason for exclusion |
| --- | --- |
| Dobson 2017 | Wrong intervention/comparison; preterm infants were started on a caffeine citrate dose of 10 mg/kg/day given once daily, and at 36 weeks postmenstrual age, infants were randomized to receive a dose of either 14 or 20 mg/kg/day given twice daily. |
| Katheria 2023 | Wrong intervention/comparison; preterm infants were randomized to the group of surfactant with caffeine citarte or caffeine citarte together with nCPAP |
| NCT00809055 2008 | Wrong intervention/comparison; high dose caffeine (caffeine citrate loading dose 40 mg/kg IV, followed 12 hours later by 20 mg/kg IV, followed 12 hours later by 10 mg/kg IV, followed 12 hours later by 10 mg/kg IV) versus standard dose caffeine (caffeine citrate loading dose 20 mg/kg IV, followed 12 hours later with D5W placebo, followed 12 hours later with 10 mg/kg IV caffeine citrate, followed 12 hours later with D5W placebo). |
| NCT03340727 2017 | Wrong intervention/comparison; caffeine (10 mg/kg/day caffeine citrate once daily, given orally before hospital discharge and 28 days after discharge) versus placebo. |
| NCT06416956 2023 | Not a randomized control trial; A prospective cohort studying preterm infants receiving caffeine as prescribed clinically. |
| Srinivasan 2002 | Wrong intervention/comparison; infants were blindly randomized to receive a bolus of either caffeine 10 mg/kg or a placebo, given once daily, followed by the alternate therapy. |

### References to excluded studies

**Dobson 2017**

- Dobson NR, Rhein LM, Darnall RA, Corwin MJ, Heeren TC, Eichenwald E, James LP, McEntire BL, Hunt CE. Caffeine decreases intermittent hypoxia in preterm infants nearing term-equivalent age. J Perinatol 2017;37(10):1135-1140. [DOI: [https://dx.doi.org/10.1038/jp.2017.82](https://doi.org/https:/dx.doi.org/10.1038/jp.2017.82)]

**Katheria 2023**

- Ines F, Hutson S, Coughlin K, Hopper A, Banerji A, Uy C, Finer N, Rich W, Morales A, Steen J, Katheria AC. Multicentre, randomised trial of preterm infants receiving caffeine and less invasive surfactant administration compared with caffeine and early continuous positive airway pressure (CaLI trial): study protocol. BMJ Open 2021;11(1):e038343-. [DOI: [https://dx.doi.org/10.1136/bmjopen-2020-038343](https://doi.org/https:/dx.doi.org/10.1136/bmjopen-2020-038343)]
- Katheria A, Ines F, Banerji A, Hopper A, Uy C, Chundu A, Coughlin K, Hutson S, Morales A, Sauberan J, Poeltler D, Dorner R, Rich W, Finer N. Caffeine and Less Invasive Surfactant Administration for Respiratory Distress Syndrome of the Newborn. NEJM Evid 2023;2(12):EVIDoa2300183-. [DOI: [https://dx.doi.org/10.1056/EVIDoa2300183](https://doi.org/https:/dx.doi.org/10.1056/EVIDoa2300183)]
- NCT04209946. Early Caffeine and LISA Compared to Caffeine and CPAP in Preterm Infants. https://clinicaltrials.gov/show/NCT04209946 2019.

**NCT00809055 2008**

- MRI and Neurodevelopment in Preterm Infants Following Administration of High-Dose Caffeine. https://clinicaltrials.gov/show/NCT00809055 2008.

**NCT03340727 2017**

- Moderately Preterm Infants With Caffeine at Home for Apnea (MoCHA) Trial. https://clinicaltrials.gov/ct2/show/NCT03340727 2017.

**NCT06416956 2023**

- NCT06416956. Baby-CINO: CaffeINe Treatment Optimisation in Premature Infants. Baby-CINO: CaffeINe Treatment Optimisation in Premature Infants 2023.

**Srinivasan 2002**

- Srinivasan P, Katz S, DeCristofaro J. Increased caffeine levels do not reduce the frequency of clinical cardiopulmonary events in neonates with apnea of prematurity following repeat bolus therapy. A randomized placebo controlled study. In: Neonatology - abstract book. Vol. 51. 2002:420A.

### Characteristics of ongoing studies

| CTRI/2023/07/055443 2023 | |
| --- | --- |
| Methods | Study design: Randomized controlled trial |
| Participants | Inclusion criteria: - Gestation less than/equal to 32 weeks - Receiving caffeine citrate (intravenous or oral), as started by the treating team, irrespective of the indication for starting caffeine - Postnatal age >= 10 days with no change in caffeine dose in the previous 72 hrs (this criterion has been introduced as there may be rapid dose changes in the initial days of life before the dose stabilizes) Exclusion criteria: - Life threatening congenital malformations (as they may not live long enough to observe apneas) Number randomized/Total sample size: Target sample size: 220 |
| Interventions | Intervention Number of participants allocated: NR Dose: NR Route of administration: NR Frequency of caffeine administration (once or twice daily): NR co-intervention (surfactant or other respiratory drugs): NR Control Number of participants allocated: NR Dose: NR Route of administration: NR Frequency of caffeine administration (once or twice daily): NR co-intervention (surfactant or other respiratory drugs): NR |
| Outcomes | Intermittent hypoxemia (IH) number of infants with at least one episode Outcome type: DichotomousOutcome Reported as: Number of participants with event (n, N) Direction: Lower is better Data value: Endpoint Chronic lung disease CLD/BPD Outcome type: DichotomousOutcome Reported as: Number of participants with event (n, N) Direction: Lower is better Data value: Endpoint Any adverse event leading to caffeine cessation Outcome type: DichotomousOutcome Reported as: Number of participants with event (n, N) Direction: Lower is better Data value: Endpoint Severe intraventricular hemorrhage (grade III and IV) Outcome type: DichotomousOutcome Reported as: Number of participants with event (n, N) Direction: Lower is better Data value: Endpoint Apnea: number of infants with at least one episode Outcome type: DichotomousOutcome Reported as: Number of participants with event (n, N) Direction: Lower is better Data value: Endpoint |
| CTRI/2023/09/057903 2023 | |
| Methods | Study design: Randomized controlled trial |
| Participants | Inclusion criteria: Inborn neonates born at completed gestational age =26 0/7 to 31 6/7 weeks, requiring invasive or non-invasive respiratory support in first 7 days of life Exclusion criteria: Neonates on invasive ventilation at time of primary outcome assessment IVH (Grade = III as per Papile Grading classification) Major congenital malformations Moribund neonates Neonates with pulmonary hypertension Number randomized/Total sample size: Target sample size: 110 |
| Interventions | Intervention Number of participants allocated: NR Dose: 5mg/kg/day to 8mg/kg/day, till 34 weeks of gestation or baby will be under room air for 7 days whichever comes first Route of administration: po Frequency of caffeine administration (once or twice daily): NR co-intervention (surfactant or other respiratory drugs): NR Control Number of participants allocated: NR Dose: standard dose of maintenance dose of caffeine: in this group maintenance dose of caffeine will remain same for the whole duration of caffeine administration and SpO2 will be measured and compared with the escalation group outcome Route of administration: po Frequency of caffeine administration (once or twice daily): NR co-intervention (surfactant or other respiratory drugs): NR |
| Outcomes | Intermittent hypoxemia (IH) number of infants with at least one episode Outcome type: DichotomousOutcome Reported as: Number of participants with event (n, N) Data value: Endpoint |

### References to ongoing studies

**CTRI/2023/07/055443 2023**

- To determine if apnea episodes(cessation of breathing in premature newborns) occur less frequently in infants whose caffeine(drug used for treatment of apnea) doses are increased for weight gain. https://trialsearch.who.int/Trial2.aspx?TrialID=CTRI/2023/07/055443 2023.

**CTRI/2023/09/057903 2023**

- Effect of increasing caffeine intake compared to usual maintenance dose in preterm neonates: a Randomised Controlled Trial. https://trialsearch.who.int/Trial2.aspx?TrialID=CTRI/2023/09/057903 2023.

### Risk of bias of included studies

| **Bias** | | |
| --- | --- | --- |
| **Domain** | **Authors' judgement** | **Support for judgement** |
| **Dani 2023** | | |
| **Risk of bias for analysis 1.1 All-cause mortality until hospital discharge** | | |
| Bias arising from the randomization process | Low risk of bias | The allocation sequence was generated by computer-generated random numbers (e-clintrials platform) that managed by statistician (one of the authors) to allow allocation concealment from investigators. The baseline clinical characteristics were similar between the two groups |
| Bias due to deviations from intended interventions | Some concerns | Participants were infants, so they were unaware of their assigned intervention. The difference in the method of drug administration between the two groups allow carers and people delivering the interventions to be aware of participants' assigned intervention during the trial |
| Bias due to missing outcome data | Low risk of bias | Data are available for all the study participants. |
| Bias in measurement of the outcome | Low risk of bias | This outcome is objectively measured. It is appropriate and similar in the two groups. No information whether the outcome assessors were aware of the intervention received by study participants or not. This outcome is observer-reported outcome that does not involve judgement, so it is unlikely to be influenced by the knowledge of the assigned intervention |
| Bias in selection of the reported results | Low risk of bias | Reported results seems to be completed |
| Overall bias | Some concerns | As explained in each domain “Support for judgement” |
| **Risk of bias for analysis 1.2 Chronic lung disease (need for oxygen or respiratory support at 36 weeks' postmenstrual age)** | | |
| Bias arising from the randomization process | Low risk of bias | The allocation sequence was generated by computer-generated random numbers (e-clintrials platform) that managed by statistician (one of the authors) to allow allocation concealment from investigators. The baseline clinical characteristics were similar between the two groups |
| Bias due to deviations from intended interventions | Some concerns | Participants were infants, so they were unaware of their assigned intervention. The difference in the method of drug administration between the two groups allow carers and people delivering the interventions to be aware of participants' assigned intervention during the trial. All randomized individuals were analyzed. i.e., no missing outcome data |
| Bias due to missing outcome data | Low risk of bias | Data are available for all the study participants |
| Bias in measurement of the outcome | Low risk of bias | This outcome is objectively measured. It is appropriate and similar in the two groups |
| Bias in selection of the reported results | Low risk of bias | Reported results seems to be completed |
| Overall bias | Some concerns | As explained in each domain “Support for judgement” |
| **Risk of bias for analysis 1.3 Duration of mechanical ventilation until hospital discharge** | | |
| Bias arising from the randomization process | Low risk of bias | The allocation sequence was generated by computer-generated random numbers (e-clintrials platform) that managed by statistician (one of the authors) to allow allocation concealment from investigators. The baseline clinical characteristics were similar between the two groups |
| Bias due to deviations from intended interventions | Some concerns | Participants were infants, so they were unaware of their assigned intervention. The difference in the method of drug administration between the two groups allow carers and people delivering the interventions to be aware of participants' assigned intervention during the trial |
| Bias due to missing outcome data | Low risk of bias | Data are available for all the study participants |
| Bias in measurement of the outcome | Low risk of bias | This outcome is objectively measured. It is appropriate and similar in the two groups. No information whether the outcome assessors were aware of the intervention received by study participants or not. The outcome is observer-reported outcome that does not involve judgement, so it is unlikely to be influenced by the knowledge of the assigned intervention |
| Bias in selection of the reported results | Low risk of bias | Reported results seems to be completed |
| Overall bias | Some concerns | As explained in each domain “Support for judgement” |
| **Risk of bias for analysis 1.4 Duration of hospital stay until hospital discharge** | | |
| Bias arising from the randomization process | Low risk of bias | The allocation sequence was generated by computer-generated random numbers (e-clintrials platform) that managed by statistician (one of the authors) to allow allocation concealment from investigators. The baseline clinical characteristics were similar between the two groups |
| Bias due to deviations from intended interventions | Some concerns | Participants were infants, so they were unaware of their assigned intervention. The difference in the method of drug administration between the two groups allow carers and people delivering the interventions to be aware of participants' assigned intervention during the trial |
| Bias due to missing outcome data | Low risk of bias | Data are available for all the study participants |
| Bias in measurement of the outcome | Low risk of bias | This outcome is objectively measured. It is appropriate and similar in the two groups. No information whether the outcome assessors were aware of the intervention received by study participants or not. The outcome is observer-reported outcome that does not involve judgement, so it is unlikely to be influenced by the knowledge of the assigned intervention |
| Bias in selection of the reported results | Low risk of bias | Reported results seems to be completed |
| Overall bias | Some concerns | As explained in each domain “Support for judgement” |
| **Risk of bias for analysis 1.5 Severe intraventricular hemorrhage until hospital discharge** | | |
| Bias arising from the randomization process | Low risk of bias | The allocation sequence was generated by computer-generated random numbers (e-clintrials platform) that managed by statistician (one of the authors) to allow allocation concealment from investigators. The baseline clinical characteristics were similar between the two groups |
| Bias due to deviations from intended interventions | Some concerns | Participants were infants, so they were unaware of their assigned intervention. The difference in the method of drug administration between the two groups allow carers and people delivering the interventions to be aware of participants' assigned intervention during the trial |
| Bias due to missing outcome data | Low risk of bias | Data are available for all the study participants |
| Bias in measurement of the outcome | Low risk of bias | This outcome is objectively measured. It is appropriate and similar in the two groups. No information whether the outcome assessors were aware of the intervention received by study participants or not. The outcome is observer-reported outcome that does not involve judgement, so it is unlikely to be influenced by the knowledge of the assigned intervention |
| Bias in selection of the reported results | Low risk of bias | Reported results seems to be completed |
| Overall bias | Some concerns | As explained in each domain “Support for judgement” |
| **Faramarzi 2018** | | |
| **Risk of bias for analysis 2.1 All-cause mortality until hospital discharge** | | |
| Bias arising from the randomization process | Some concerns | It is an open-label randomized clinical trial. The only information about randomization methods is the statement that participants were randomly assigned to the study groups. No information that the allocation sequence was concealed until participants were enrolled and assigned to interventions. No significant differences or excessive similarity between the two groups in the baseline characteristics |
| Bias due to deviations from intended interventions | Some concerns | It is an open-label randomized clinical trial. Participants were infants so they were unaware of their assigned intervention during the trial |
| Bias due to missing outcome data | Low risk of bias | Data are available for all the study participants |
| Bias in measurement of the outcome | Low risk of bias | The outcome assessor judgement is negligible |
| Bias in selection of the reported results | Low risk of bias | Reported results seems to be completed |
| Overall bias | Some concerns | As explained in each domain “Support for judgement” |
| **Risk of bias for analysis 2.2 Duration of mechanical ventilation until hospital discharge** | | |
| Bias arising from the randomization process | Some concerns | It is an open-label randomized clinical trial. The only information about randomization methods is the statement that participants were randomly assigned to the study groups. No information that the allocation sequence was concealed until participants were enrolled and assigned to interventions. No significant differences or excessive similarity between the two groups in the baseline characteristics |
| Bias due to deviations from intended interventions | Some concerns | It is an open-label randomized clinical trial. Participants were infants so they were unaware of their assigned intervention during the trial |
| Bias due to missing outcome data | Low risk of bias | Data are available for all the study participants |
| Bias in measurement of the outcome | Low risk of bias | The outcome assessor judgement is negligible |
| Bias in selection of the reported results | Low risk of bias | Reported results seems to be completed |
| Overall bias | Some concerns | As explained in each domain “Support for judgement” |
| **Risk of bias for analysis 2.3 hospital stay until hospital discharge** | | |
| Bias arising from the randomization process | Some concerns | It is an open-label randomized clinical trial. The only information about randomization methods is the statement that participants were randomly assigned to the study groups. No information that the allocation sequence was concealed until participants were enrolled and assigned to interventions. No significant differences or excessive similarity between the two groups in the baseline characteristics |
| Bias due to deviations from intended interventions | Some concerns | It is an open-label randomized clinical trial. Participants were infants so they were unaware of their assigned intervention during the trial |
| Bias due to missing outcome data | Low risk of bias | Data are available for all the study participants |
| Bias in measurement of the outcome | Low risk of bias | The outcome assessor judgement is negligible |
| Bias in selection of the reported results | Low risk of bias | Reported results seems to be completed |
| Overall bias | Some concerns | As explained in each domain “Support for judgement” |

### Analyses

#### *Analysis 1: Intravenous versus oral administration of the initial caffeine dose in preterm infants*

| Outcome | No. of studies (participants) | Statistical method | Effect size |
| --- | --- | --- | --- |
| 1.1 All-cause mortality until hospital discharge | 1 (38) | Risk Ratio (M-H, Fixed, 95% CI) | 0.14 [0.01, 2.59] |
| 1.2 Chronic lung disease (need for oxygen or respiratory support at 36 weeks' postmenstrual age) | 1 (38) | Risk Ratio (M-H, Fixed, 95% CI) | 5.00 [0.26, 97.70] |
| 1.3 Duration of mechanical ventilation | 1 (38) | Mean Difference (IV, Fixed, 95% CI) | 1.00 [-8.05, 10.05] |
| 1.4 Duration of hospital stay | 1 (38) | Mean Difference (IV, Fixed, 95% CI) | 8.00 [-5.35, 21.35] |
| 1.5 Severe intraventricular hemorrhage until hospital discharge | 1 (38) | Risk Ratio (M-H, Fixed, 95% CI) | 1.00 [0.29, 3.43] |

#### *Analysis 2: Twice-daily (split dose) caffeine administration vs once-daily caffeine administration in preterm infants*

| Outcome | No. of studies  (participants) | Statistical method | Effect size |
| --- | --- | --- | --- |
| 2.1 All-cause mortality until hospital discharge | 1 (40) | Risk Ratio (M-H, Fixed, 95% CI) | 1.00 [0.29, 3.45] |
| 2.2 Duration of mechanical ventilation | 1 (40) | Mean Difference (IV, Fixed, 95% CI) | 0.81 [-7.56, 9.18] |
| 2.3 Duration of hospital stay | 1 (40) | Mean Difference (IV, Fixed, 95% CI) | -2.70 [-8.12, 2.72] |

### Figures: forest plots

Figura 1: Analysis 1.1

Figura 2: Analysis 1.2

Figura 3: Analysis 1.3

Figura 4: Analysis 1.4

Figura 5: Analysis 1.5

Figura 6: Analysis 2.1

Figura 7: Analysis 2.2

Figura 8: Analysis 2.3
